# Notch4 and mhc class II polymorphisms are associated with hcv-related benign and malignant lymphoproliferative diseases

**DOI:** 10.18632/oncotarget.17655

**Published:** 2017-05-06

**Authors:** Laura Gragnani, Elisa Fognani, Valli De Re, Massimo Libra, Adriana Garozzo, Patrizio Caini, Guia Cerretelli, Andrea Giovannelli, Serena Lorini, Monica Monti, Silvia Bagnoli, Irene Piaceri, Anna Linda Zignego

**Affiliations:** ^1^ Center for Systemic Manifestations of Hepatitis Viruses (MaSVE), Department of Experimental and Clinical Medicine, University of Florence, Florence, Italy; ^2^ Centro di Riferimento oncologico, National Cancer Institute, Aviano, Italy; ^3^ Department of Biomedical and Biotechnological Sciences, Section of Microbiology, University of Catania, Italy; ^4^ Department of Neuroscience, Psychology, Drug Research and Children's Health, University of Florence, Florence, Italy

**Keywords:** HCV, mixed cryoglobulinemia, NOTCH4, HLA-II, lymphoma

## Abstract

Mixed cryoglobulinemia (MC), is a HCV-related, clinically benign, lymphoproliferative disorder (LPD) that may evolve into a non Hodgkin's lymphoma (NHL). Significant associations were found between two single nucleotide polymorphisms near NOTCH4 (rs2071286) and the HLA class II (rs9461776) genes and HCV-related MC syndrome (MCS). We analyzed NOTCH4 rs2071286 and HLA-II rs9461776 in 3 HCV-related LPD groups (asymptomatic MC, MCS, NHL) with HCV infection without lymphoproliferative disorders.

We found a positive relationship between NOTCH4 rs207186 T minor allele frequency (MAF) and patients with HCV-related LPDs at risk of NHL (Chi-square test for trend = 14.84 *p* = 0.0001), in accordance with an over-dominant model in the NHL group (CT vs CC + TT, OR=1.88, 95% CI 1.24–2.83, *p* = 0.0026).

Regarding HLA II rs9461776, G MAF increased in patients with HCV-related LPDs at risk of NHL (Chi-square test for trend = 8.40 *p* = 0.0038), in accordance with a recessive genotypic model in the NHL group (G/G vs A/A + A/G, OR = 11.07, 95% CI 2.37–51.64, *p* = 0.0022).

Both NOTCH4 rs2071286 and HLA-II rs9461776 were present on chromosome 6 and showed D’ and r values of linkage disequilibrium (LD) of about 0.5 values, thereby suggesting there is no extensive LD in the HCV+ population.

This data shows that the previously demonstrated association between NOTCH4 rs2071286 and HLA-II rs9461776 polymorphisms and HCV-related MCS could be extended to overall patients with HCV-related LPDs. The significant relationship between rs2071286 and rs9461776 MAF and the increased risk for NHL, suggests their use as non-invasive markers to categorize patients at risk of lymphoma.

## INTRODUCTION

Hepatitis C Virus (HCV) has a high propensity to persist in the host, leading to chronic liver disease but it is also known that in a smaller percentage of patients it can cause lymphoproliferative disorders (LPDs) [1]: the most frequent one is called mixed cryoglobulinemia (MC) [2, 3]. MC is a lymphoproliferative/autoimmune disease, clinically benign, with an increased risk of developing B cell Non-Hodgkin lymphoma (NHL) [1, 4, 5]. MC is characterized by the presence of circulating immune-complexes called cryoglobulins (CGs). Some patients exhibit a symptomatic MC called MC syndrome (MCS) or MC Vasculitis which derives from a systemic involvement of small, medium-sized vessels; other subjects do not show any symptoms even if they have CGs in their serum. The relationship between NHL and HCV has now been confirmed in a large number of studies with the most convincing evidence resulting from the reduced incidence of NHL in patients after successful HCV eradication [6] and by using different transgenic murine models [7, 8]. However, the lymphoma genetic mechanism related to HCV infection still has to be clarified. Several models have been proposed not all mutually exclusive, such as a direct oncogenic effect or an antigen driven lymphocyte proliferation [1, 9–11]. A rational hypothesis could be a specific favorable host's genetics that increases the susceptibility to LPDs when the virus persists. While there was not clear and convincing evidence to sustain a role of particular HCV genotypes, the differing prevalence of HCV-related LPDs in different geographical areas [12] seems to confirm the importance of host genetics predisposed to LPDs [13–17]. Several genetic studies had focused their attention on the human leukocyte antigen (HLA) variants highlighting an association between specific classes of genes and the susceptibility to the development of HCV-related MC and NHL [18–22]. However a clear result as of today has not been obtained, due in part to the extreme polymorphic nature of the HLA loci. A multicenter Genome Wide Association Study (GWAS) showed an association of two particular polymorphisms located on chromosome 6 with HCV-related MC Vasculitis compared to HCV controls without LPDs [23]; the first one is a single nucleotide polymorphism (SNP) (rs2071286) located in an intronic region of the NOTCH4 gene, the second one is a SNP (rs9461776) located between HLA-DRB1 and HLA-DQA1 gene segments of the class II major histocompatibility complex (MHC).

NOTCH4 gene encodes for a protein belonging to the NOTCH family of transmembrane proteins with repeated extracellular epidermal growth factor-like domains and an intracellular domain [24, 25]. NOTCH signaling is involved in many biological processes, ranging from embryonic development to cell proliferation and survival and its main action is to regulate the interaction between adjacent cells through binding with its cognate ligand [26]. Notch4 is expressed in blood and human bone marrow cells –derived CD34+ progenitor cells as well as in CD34− cell population [27]. It was suggested that activation of NOTCH4 leads to enhanced stem cell activity, reduces differentiation and alters lymphocyte development [28].

Based on the GWAS results, we evaluated the allelic frequencies of the NOTCH4 rs2071286 and the HLA-II rs9461776 SNPs, in a wide cohort of patients with different HCV-related LPDs with circulating cryoglobulins, with and without vasculitis, or with HCV− related NHL, comparing them to a group of HCV+ patients without LPDs.

## RESULTS

### Patient characteristics and group distribution

Patient characteristics and group distribution were shown in Table 1.

**Table 1:** Main clinical and laboratory findings of HCV chronically infected patients according to extra-hepatic conditions

	HCV (*n* = 85)	MC-HCV (*n* = 73)	MCS-HCV (*n* = 108)	NHL-HCV (*n* = 61)
**Mean Age** (years)	52.4 ± 11.6	56.2 ± 12.5	61.5 ± 13.9	62.6 ± 7.6
**Sex** (male/female)	48/37	38/35	40/68^§^	23/38^§§^
**Viral titer** (IU/mL × 10^6^)	2.9 ± 3.1	3.3 ± 2.8	3.4 ± 6.6	3.5 ± 4.1
**HCV genotype**				
1	47 (55%)	41 (56%)	60 (55%)	52 (85%)
2	24 (28%)	17 (23%)	32 (30%)	7 (12%)
3–4	14 (17%)	15 (21%)	16 (15%)	2 (3%)
**Mean cryocrit** (%)	-	3.4 ± 5.1	11.7 ± 17.1	4.8 ± 5.4^*^
**Histology**				
Diffuse Large Cell Lymphoma				26 (43%)
Marginal Zone Lymphoma				25 (41%)
Follicular Lymphoma				4 (6.5%)
Small Lymphocytic Lymphoma				3 (5%)
MALT				2 (3%)
Burkitt lymphoma				1 (1.5%)

MC: Mixed Cryoglobulinemia; MCS: Mixed Cryoglobulinemia Syndrome; NHL: Non-Hodgkin's Lymphoma; IU: International Units; MALT: mucosa-associated lymphoid tissue;

*data were available for 33 patients out of the 61 analyzed; ^§^*p* < 0.01; ^§§^*p* < 0.05.

The groups did not significantly differ in terms of viremia titers or viral genotype distribution, whereas, as expected, the female sex was more significantly represented in the MCS-HCV group (*p <* 0.01) and NHL-HCV group (*p <* 0.05).

### Genotype frequencies

**Figure 1:**
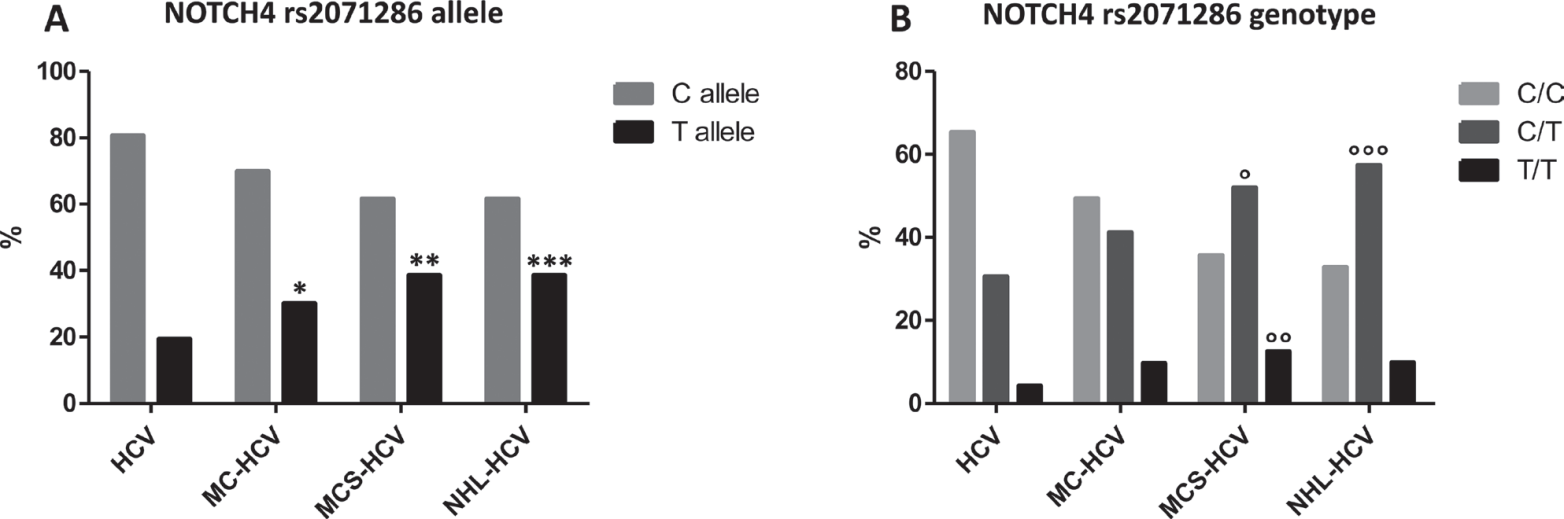
(Panel **A**) NOTCH4 rs2071286 allele frequency; *p = 0.004; **p = 0.0002; ***p = 0.0006; (Panel **B**) NOTCH4 rs2071286 genotype frequency; °*p* = 0.008; °°*p* = 0.0122; °°°*p* = 0.006.

**Figure 2:**
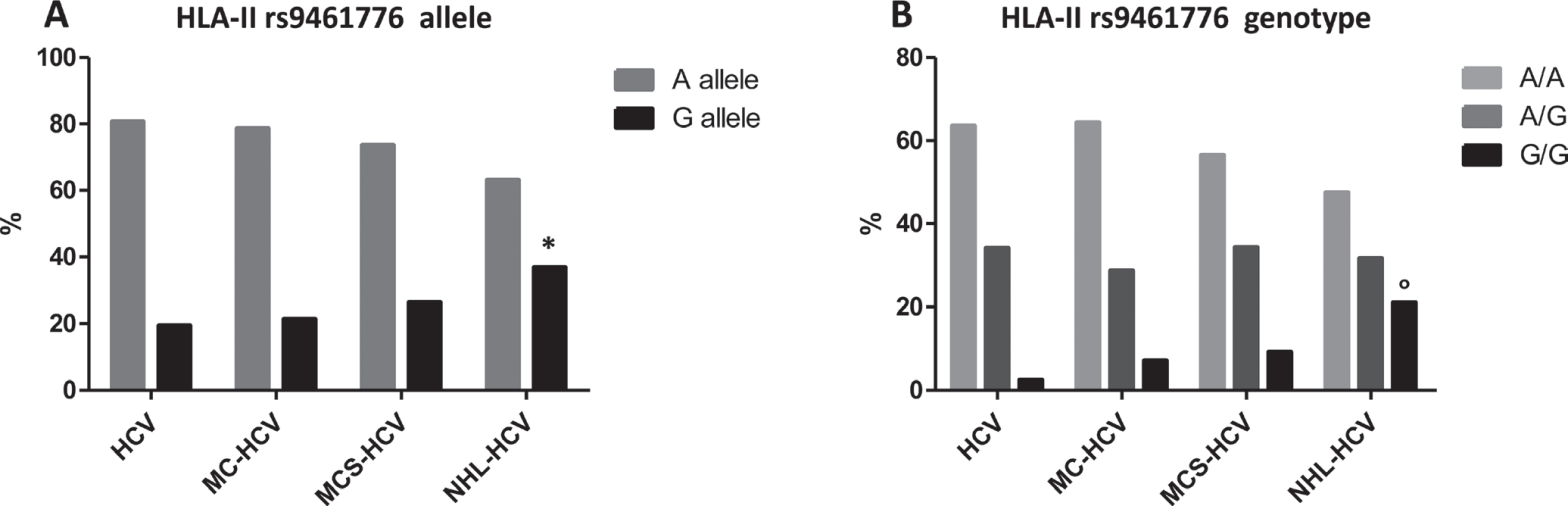
(Panel **A**) HLA-II rs9461776 allele frequency; *p = 0.0015; (Panel **B**) HLA-II rs9461776 genotype frequency; °*p* = 0.0006.

Results obtained from the analysis of allele and genotype, for NOTCH4 rs2071286 and HLA-II rs9461776 are summarized in Figure 1 and Figure 2 (allele frequency in panels A and genotype frequency in panels B).

### NOTCH4 rs2071286 SNP analysis

all the three groups of patients with HCV-related LPDs considered in this study (MC-HCV, MCS-HCV and NHL-HCV) showed a higher presence of the rs2071286 T minor allele frequency (MAF) in respect to the HCV control group (OR 1.79, 95% CI 1.04–3.08, *p* = 0.04; OR 2.59, 95% CI 1.57–4.26, *p* = 0.0002 and OR 2.6, 95% CI 1.5–4.5, *p* = 0.0006, respectively) (Figure 1, panel 1A). We found a positive relationship between T minor allele frequency (MAF) and the proportion of patients with HCV-related LPDs at risk of NHL (chi-square test for trend = 14.84, *p* = 0.0001), in accordance with an over-dominant model in NHL group (CT vs CC + TT, OR = 1.88, 95% CI 1.24–2.83, *p* = 0.0026).

### HLA rs9461776 SNP analysis

Regarding the HLA-II rs9461776 polymorphism, we found a G MAF increase in the proportion of patients with HCV-related LPDs at risk of NHL (chi-square test for trend = 8.40 *p* = 0.0038) (Figure 2, panel A), in accordance with a recessive genotypic model in the NHL group (G/G vs A/A + A/G, OR = 12, 95% CI 2.50–57.51, *p* = 0.0006).

### Linkage disequilibrium

To date, the functional role of NOTCH4 rs2071286 and HLA-II rs9461776 are unknown. In particular, it is unknown whether rs168924 or HLA-II rs9461776 are in linkage disequilibrium (LD) with another functionally significant SNP. Linkage disequilibrium between NOTCH4 rs2071286 and HLA-II rs9461776 polymorphisms was then calculated. Genotyping of our samples indicated no significant LD between the 2 SNPs (D’ = 0.5423, *r* = 0.5184), therefore NOTCH4 rs2071286 and HLA-II rs9461776 did not usually occur on the same haplotype.

### Haplotype analysis

The two SNPs have been grouped into 4 haplotypes (Table 2). The more frequent haplotype AC included 2 wild type alleles HLA-II A-rs9461776 and NOTCH4 C-rs2071286.

**Table 2:** NOTCH4 rs2071286 and HLA rs9461776 haplotype

Haplotype	HCV %	MC-HCV %	OR (95% CI) CRUDE		MCS-HCV %	OR (95% CI) CRUDE		NHL-HCV %	OR (95% CI) CRUDE	
			Overall	*p*		Overall	*p*		Overall	*p*
**AC**	75.99	65.07	1 (reference)		55.69	1 (reference)		41.15	1 (reference)	
**GT**	11.41	15.79	1.65 (0.77-3.52)	0.16	20.76	2.54 (1.30-5.01)	0.003	22.82	3.81 (1.52-9.59)	0.001
**AT**	8.03	13.5	1.90 (0.82-4.41)	0.10	17.7	2.29 (1.39-6.26)	0.002	17.18	3.63 (1.28-10.32)	0.005
**GC**	4.56	5.64	1.37 (0.43-4.39)	0.55	5.85	1.58 (0.55-4.64)	0.35	18.85	6.85 (2.18-22.10)	0.00007

MC: Mixed Cryoglobulinemia; MCS: Mixed Cryoglobulinemia Syndrome; NHL: Non-Hodgkin's Lymphoma; OR: Odds ratio; CI: confidence interval; statistical analysis was performed using X^2^ test.

The remaining haplotypes that all included at least one minor allele (GT, AT, and GC) were found associated with the NHL-HCV group of patients (OR. 3.81, *p* = 0.001,; OR. 3.63, *p* = 0.005,; OR 6.85, *p* = 0.00007, respectively). The most frequent haplotype (22.8%) associated with NHL-HCV included both the GT MAF, while the GC haplotype showed the highest OR value (6.85) denoted HLA-II G MAF may be more strongly associated than NOTCH4 C-rs2071286 with NHL-HCV condition. By converse, NOTCH4 T-rs2071286 showed the best association with MCS-HCV cases, since both GT and AT haplotypes found were significantly associated with MCS-HCV (*p* = 0.003, O.R. 2.54 and *p* = 0.002, O.R. 2.29, respectively) (Table 2).

## DISCUSSION

Two SNPs, previously associated with symptomatic MC in a multicenter GWAS study were tested. By using these SNPs we were able to define a trend for an increased risk for NHL-HCV development in patients with HCV-related LPDs. Since the Therapy Guidelines for HCV-treatment with direct acting antiviral drugs (DAAs) are stringent (http://www.easl.eu/medias/cpg/HCV2016/English-report.pdf), an easy way to recognize subjects at risk of NHL may be helpful to evaluate the treatment priority.

The HLA region is well known for being associated with different autoimmune and infectious diseases [29, 30].

Both the SNPs tested in the present study lie in non-coding regions, suggesting that SNPs had either a regulatory role or were indirect tag-SNPs useful to identifying functional and until today unknown mutations involved in the HCV-related LPDs process. The medium LD between NOTCH4 rs2071286 and HLA-II rs9461776 polymorphisms suggested that a functional sequence included in the NOTCH4 and HLA-II region, could have a role in HCV-related LPDs. A potential role of NOTCH4 in the pathogenesis of HCV-related LPDs and members of the NOTCH family in several malignancies such as leukemia, gastric breast, bladder, ovarian and colon cancer were consistent with a role of NOTCH protein alteration, in the onset of some autoimmune disorders including NHL-HCV malignancies [31–34]. Moreover, Arcaini and co-workers observed that NOTCH pathway somatic mutations are associated with 25% of HCV-positive diffuse large B-cells lymphoma cases [35].

Among the notch family, NOTCH4 and its signaling is the least studied member; as already mentioned, several reports showed an association between SNPs either within or near NOTCH4 and different autoimmunities [36], but, so far, no indication about its functional role has been proposed. In fact, its expression seems to be relevant for the development of the central nervous system [37] and vascular system [38], and also appears to play a role in the differentiation of naive CD4+ T cells [39]. Interestingly, a very recent paper listed NOTCH4 among genes with a pleiotropic effect, meaning that it could be able to determine multiple phenotypic traits [40]. As other examples of pleiotropy, NOTCH4 variants could determine a shared genetic susceptibility mechanism between different diseases or physiological processes. in this light, it is not surprising that a gene, so far implied in the biology of development, could also be involved in the development of autoimmunities and haematological malignancies.

Our data also suggested that HLA-II region represented a strong susceptibility area for several HCV-related LPDs, in accordance with previous studies [41, 42].

In conclusion, the present study not only confirmed the association of NOTCH4 rs2071286 and HLA-II rs9461776 with HCV-MCS, but indicated these SNPs as potential markers for HCV-related LPDs susceptibility, in particular with an increased risk for HCV-NHL development. These observations should be confirmed in a wider population before suggesting the use of this SNP as a biomarker at a higher risk of developing hematological malignancies.

Nonetheless, together with the evidence of previously published results regarding these SNPs in HCV-related disorders, our study highlights the interest for a deeper molecular analysis aimed at clarifying the functional role of genes included among NOTCH4 and the HLA-II region gene that increases the odds of developing a NHL during HCV-chronic infection.

## MATERIALS AND METHODS

This is a retrospective case-control study; patients were recruited from April 2009 to January 2016 in three different Italian Centers: Center for Systemic Manifestations of Hepatitis Viruses (MaSVE) (Department of Experimental and Clinical Medicine, University of Florence, Florence); Centro di Riferimento Oncologico, National Cancer Institute, Aviano, Italy and the Department of Biomedical and Biotechnological Sciences, University of Catania, Italy.

Peripheral blood samples from 327 HCV-infected patients, grouped as follows: 85 patients without any evidence of serum cryoglobulins (CGs) or autoimmune/lymphoproliferative disorders (HCV group), 73 with circulating CGs but without symptoms of vasculitis (MC-HCV group), 108 with HCV-related CGs and vasculitis (MCS-HCV) and 61 with HCV-related NHL (NHL-HCV) were analyzed.

The ethnicity of all the patients was Caucasian. Patients positive at the HBV and/or the HIV test were excluded from the study. Demographic and clinical characteristics of each groups are reported on Table 1.

HCV infection was proven by detecting circulating anti-HCV antibodies (EIA-2 and RIBA-2; Ortho Diagnostic Systems, Raritan, NJ) and HCV RNA (AMPLICOR HCV Test, v2.0; Roche Diagnostics, Alameda, CA). The HCV genotype was assessed by the VERSANT HCV Genotype 2.0 assay (Siemens Healthcare Diagnostics, Deerfield, IL).

Mixed cryoglobulinemia was assessed by circulating CGs found in at least three metachronous samples. All patients with MCS satisfied available classification criteria. [43, 44]. NHL type was defined according to updated WHO classification of hematological malignancies [45].

The study was conducted in accordance with the ethical guidelines of the Helsinki Declaration. All subjects provided written informed consent and the protocol was approved by independent local ethics committees.

### Cell isolation and DNA extraction

Peripheral blood mononuclear cells (PBMCs) were isolated from fresh anticoagulated blood by gradient precipitation on Lymphoprep (Axis-Shield PoC AS, Oslo, Norway) according to the manufacturer's instructions. After the second wash, the cells were counted and stored at −80°C.

Genomic DNA was extracted from PBMCs using an QIAamp DNA Mini Kit (QIAGEN Inc, Valencia, CA, USA) or from whole blood samples using a Nucleospin Blood Kit (Macherey-Nagel GmbH & Co. KG, Düren, Germany) according to the manufacturer's instructions.

### SNPs genotyping

The rs2071286 (NOTCH4) and the rs9461776 (HLA) SNP genotyping was performed using a specific TaqMan SNP Genotyping Assay (Applied Biosystem, Foster City, CA, USA) with supplied probes and primers, on a Rotor Gene 6000 (Corbett Research, Sidney, Australia).

### Statistical analysis

Statistical analysis was performed using SPSS statistical software, version 23 (SPSS, Inc. Chicago, IL, USA). Comparisons of genotype and allele frequencies were performed using the X^2^ test. The haplotypes frequencies were estimated using PowerMarker software, version 3.25 (http://statgen.ncsu.edu/powermarker/), and the obtained data was arranged in a contingency table. Comparisons of haplotype frequencies were performed using the X^2^ test without confounding variables. Linkage disequilibrium between SNPs was analyzed using PowerMarker software, version 3.25 as well. All tests were two-sided at a 0.05 significance level.

## Abbreviations

MC: mixed cryoglobulinemia
NHL: non-Hodgkin's lymphoma
LPD: lymphoproliferative disorder
MCS: mixed cryoglobulinemia syndrome
GWAS: genome wide association study
MAF: minor allele frequency
HLA: human leukocyte antigen
CGs: cryoglobulins
LD: linkage disequilibrium
PBMCs: peripheral blood mononuclear cells

## References

[1] Cacoub P, Gragnani L, Comarmond C, Zignego AL (2014). Extrahepatic manifestations of chronic hepatitis C virus infection. Dig Liver Dis.

[2] Zignego AL, Gragnani L, Piluso A, Sebastiani M, Giuggioli D, Fallahi P, Antonelli A, Ferri C (2015). Virus-driven autoimmunity and lymphoproliferation: the example of HCV infection. Expert Rev Clin Immunol.

[3] Ferri C, La Civita L, Longombardo G, Lombardini F, Pasero G, Zignego AL, Monti M, Mazzaro C, Greco F, Mazzoni A (1994). Hepatitis C virus in mixed cryoglobulinemia and B cell lymphoma [letter]. Clin Exp Rheumatol.

[4] Ferri C, La Civita L, Caracciolo F, Zignego AL (1994). Non-Hodgkin's lymphoma: possible role of hepatitis C virus [letter]. Jama.

[5] Monti G, Pioltelli P, Saccardo F, Campanini M, Candela M, Cavallero G, De Vita S, Ferri C, Mazzaro C, Migliaresi S, Ossi E, Pietrogrande M, Gabrielli A (2005). Incidence and characteristics of non-Hodgkin lymphomas in a multicenter case file of patients with hepatitis C virus-related symptomatic mixed cryoglobulinemias. Arch Intern Med.

[6] Kawamura Y, Ikeda K, Arase Y, Yatsuji H, Sezaki H, Hosaka T, Akuta N, Kobayashi M, Suzuki F, Suzuki Y, Kumada H (2007). Viral elimination reduces incidence of malignant lymphoma in patients with hepatitis C. Am J Med.

[7] Machida K, Tsukiyama-Kohara K, Sekiguch S, Seike E, Tone S, Hayashi Y, Tobita Y, Kasama Y, Shimizu M, Takahashi H, Taya C, Yonekawa H, Tanaka N (2009). Hepatitis C virus and disrupted interferon signaling promote lymphoproliferation via type II CD95 and interleukins. Gastroenterology.

[8] Kasama Y, Sekiguchi S, Saito M, Tanaka K, Satoh M, Kuwahara K, Sakaguchi N, Takeya M, Hiasa Y, Kohara M, Tsukiyama-Kohara K (2010). Persistent expression of the full genome of hepatitis C virus in B cells induces spontaneous development of B-cell lymphomas in vivo. Blood.

[9] Peveling-Oberhag J, Arcaini L, Hansmann ML, Zeuzem S (2013). Hepatitis C-associated B-cell non-Hodgkin lymphomas. Epidemiology, molecular signature and clinical management. J Hepatol.

[10] Zignego AL, Giannini C, Gragnani L (2012). HCV and lymphoproliferation. Clin Dev Immunol.

[11] De Re V, De Vita S, Marzotto A, Rupolo M, Gloghini A, Pivetta B, Gasparotto D, Carbone A, Boiocchi M (2000). Sequence analysis of the immunoglobulin antigen receptor of hepatitis C virus-associated non-Hodgkin lymphomas suggests that the malignant cells are derived from the rheumatoid factor-producing cells that occur mainly in type II cryoglobulinemia. Blood.

[12] Pozzato G, Burrone O, Baba K, Matsumoto M, Hijiiata M, Ota Y, Mazzoran L, Baracetti S, Zorat F, Mishiro S, Efremov DG (1999). Ethnic difference in the prevalence of monoclonal B-cell proliferation in patients affected by hepatitis C virus chronic liver disease. J Hepatol.

[13] Persico M, Capasso M, Persico E, Masarone M, Renzo A, Spano D, Bruno S, Iolascon A (2006). Interleukin-10 - 1082 GG polymorphism influences the occurrence and the clinical characteristics of hepatitis C virus infection. J Hepatol.

[14] Fabris M, Quartuccio L, Salvin S, Pozzato G, De Re V, Mazzaro C, Ferri C, Baldini C, De Vita S (2008). Fibronectin gene polymorphisms are associated with the development of B-cell lymphoma in type II mixed cryoglobulinemia. Ann Rheum Dis.

[15] Giannini C, Gragnani L, Piluso A, Caini P, Petrarca A, Monti M, Laffi G, Zignego AL (2008). Can BAFF promoter polymorphism be a predisposing condition for HCV-related mixed cryoglobulinemia?. Blood.

[16] Gragnani L, Piluso A, Giannini C, Caini P, Fognani E, Monti M, Petrarca A, Ranieri J, Razzolini G, Froio V, Laffi G, Zignego AL (2011). Genetic determinants in hepatitis C virus-associated mixed cryoglobulinemia: role of polymorphic variants of BAFF promoter and Fcgamma receptors. Arthritis Rheum.

[17] Farawela H, Khorshied M, Shaheen I, Gouda H, Nasef A, Abulata N, Mahmoud HA, Zawam HM, Mousa SM (2012). The association between hepatitis C virus infection, genetic polymorphisms of oxidative stress genes and B-cell non-Hodgkin lymphoma risk in Egypt. Infect Genet Evol.

[18] Ossi E, Bordin MC, Businaro MA, Marson P, Bonadonna P, Chiaramonte M, Boin F, Valenti MT, Fagiolo U (1995). HLA expression in type II mixed cryoglobulinemia and chronic hepatitis C virus. Clin Exp Rheumatol.

[19] Lenzi M, Frisoni M, Mantovani V, Ricci P, Muratori L, Francesconi R, Cuccia M, Ferri S, Bianchi FB (1998). Haplotype HLA-B8-DR3 confers susceptibility to hepatitis C virus- related mixed cryoglobulinemia. Blood.

[20] Amoroso A, Berrino M, Canale L, Cornaglia M, Guarrera S, Mazzola G, Savoldi S, Scolari F, Sallberg M, Clementi M, Gabrielli A (1998). Are HLA class II and immunoglobulin constant region genes involved in the pathogenesis of mixed cryoglobulinemia type II after hepatitis C virus infection?. J Hepatol.

[21] Congia M, Clemente MG, Dessi C, Cucca F, Mazzoleni AP, Frau F, Lampis R, Cao A, Lai ME, De Virgiliis S (1996). HLA class II genes in chronic hepatitis C virus-infection and associated immunological disorders. Hepatology.

[22] De Re V, Caggiari L, Monti G, Libra M, Spina M, Dolcetti R, De Zorzi M, Racanelli V, Crovatto M, Toffoli G (2010). HLA DR-DQ combination associated with the increased risk of developing human HCV positive non-Hodgkin lymphoma is related to the type II mixed cryoglobulinemia. Tissue Antigens.

[23] Zignego AL, Wojcik GL, Cacoub P, Visentini M, Casato M, Mangia A, Latanich R, Charles ED, Gragnani L, Terrier B, Piazzola V, Dustin LB, Khakoo SI (2014). Genome-wide association study of hepatitis C virus- and cryoglobulin-related vasculitis. Genes Immun.

[24] D'souza MS, Markou A (2010). Neural substrates of psychostimulant withdrawal-induced anhedonia. Curr Top Behav Neurosci.

[25] Kovall RA, Blacklow SC (2010). Mechanistic insights into Notch receptor signaling from structural and biochemical studies. Curr Top Dev Biol.

[26] Pancewicz J, Nicot C (2011). Current views on the role of Notch signaling and the pathogenesis of human leukemia. BMC Cancer.

[27] Karanu FN, Yuefei L, Gallacher L, Sakano S, Bhatia M (2003). Differential response of primitive human CD34- and CD34+ hematopoietic cells to the Notch ligand Jagged-1. Leukemia.

[28] Vercauteren SM, Sutherland HJ (2004). Constitutively active Notch4 promotes early human hematopoietic progenitor cell maintenance while inhibiting differentiation and causes lymphoid abnormalities in vivo. Blood.

[29] Zanelli E, Breedveld FC, de Vries RR (2000). HLA association with autoimmune disease: a failure to protect?. Rheumatology (Oxford).

[30] Sirota M, Schaub MA, Batzoglou S, Robinson WH, Butte AJ (2009). Autoimmune disease classification by inverse association with SNP alleles. PLoS Genet.

[31] Epenetos AA, Kousparou C, Stylianou S (2009). Inhibition of Notch and tumor regression. Journal of Clinical Oncology.

[32] Hopfer O, Zwahlen D, Fey MF, Aebi S (2005). The Notch pathway in ovarian carcinomas and adenomas. Br J Cancer.

[33] Yousif NG, Deb AA, Al-Matwari M, Mousa HJ (2012). Prognostic impact of expression Notch-1 in invasive bladder transitional cell carcinoma. Journal of Clinical Oncology.

[34] Huang TT, Zhou YH, Cheng ASL, Yu J, To KF, Kang W (2016). NOTCH receptors in gastric and other gastrointestinal cancers: oncogenes or tumor suppressors?. Molecular Cancer.

[35] Arcaini L, Rossi D, Lucioni M, Nicola M, Bruscaggin A, Fiaccadori V, Riboni R, Ramponi A, Ferretti VV, Cresta S, Casaluci GM, Bonfichi M, Gotti M (2015). The NOTCH pathway is recurrently mutated in diffuse large B-cell lymphoma associated with hepatitis C virus infection. Haematologica.

[36] Morris DL, Taylor KE, Fernando MM, Nititham J, Alarcon-Riquelme ME, Barcellos LF, Behrens TW, Cotsapas C, Gaffney PM, Graham RR, Pons-Estel BA, Gregersen PK, Harley JB (2012). Unraveling multiple MHC gene associations with systemic lupus erythematosus: model choice indicates a role for HLA alleles and non-HLA genes in Europeans. Am J Hum Genet.

[37] Grandbarbe L, Bouissac J, Rand M, Hrabe de Angelis M, Artavanis-Tsakonas S, Mohier E (2003). Delta-Notch signaling controls the generation of neurons/glia from neural stem cells in a stepwise process. Development.

[38] Murphy PA, Lam MT, Wu X, Kim TN, Vartanian SM, Bollen AW, Carlson TR, Wang RA (2008). Endothelial Notch4 signaling induces hallmarks of brain arteriovenous malformations in mice. Proc Natl Acad Sci USA.

[39] Yamane H, Paul WE (2013). Early signaling events that underlie fate decisions of naive CD4(+) T cells toward distinct T-helper cell subsets. Immunol Rev.

[40] Zheng W, Rao S (2015). Knowledge-based analysis of genetic associations of rheumatoid arthritis to inform studies searching for pleiotropic genes: a literature review and network analysis. Arthritis Res Ther.

[41] De Re V, Caggiari L, Simula MP, De Vita S, Mazzaro C, Lenzi M, Massimo GM, Monti G, Ferri C, Zignego AL, Gabrielli A, Sansonno D, Dammacco F (2007). Role of the HLA class II: HCV-related disorders. Ann N Y Acad Sci.

[42] Gragnani L, Fognani E, Piluso A, Zignego AL (2013). Hepatitis C virus-related mixed cryoglobulinemia: is genetics to blame?. World J Gastroenterol.

[43] Quartuccio L, Isola M, Corazza L, Ramos-Casals M, Retamozo S, Ragab GM, Zoheir MN, El-Menyawi MA, Salem MN, Sansonno D, Ferraccioli G, Gremese E, Tzioufas A (2014). Validation of the classification criteria for cryoglobulinaemic vasculitis. Rheumatology (Oxford).

[44] Ferri C, Ramos-Casals M, Zignego AL, Arcaini L, Roccatello D, Antonelli A, Saadoun D, Desbois AC, Sebastiani M, Casato M, Lamprecht P, Mangia A, Tzioufas AG (2016). International diagnostic guidelines for patients with HCV-related extrahepatic manifestations. A multidisciplinary expert statement. Autoimmun Rev.

[45] Swerdlow SH, Campo E, Pileri SA, Harris NL, Stein H, Siebert R, Advani R, Ghielmini M, Salles GA, Zelenetz AD, Jaffe ES (2016). The 2016 revision of the World Health Organization classification of lymphoid neoplasms. Blood.

